# Handbook of Physiology

**Published:** 1873-11

**Authors:** 


*Handbook of Physiology.* By William Senhouse Kirkes, M. D. Edited by W. Morrant Baker, F. R. C. S. With Two Hundred and forty-eight illustrations. New American from the Eighth English Edition. Philadelphia: Henry C. Lea, 1873. Buffalo: T. Butler & Son.

Kirkes Handbook of Physiology has been long and favorably known by the profession, and the increased demand for the work has called for the publication of a new edition. The present one is but little more, however, than a reprint of the last, there being but little change in the text. The discussion of certain points now considered as settled is omitted, which has reduced the size of the book a few pages, less than a dozen. A few other changes have been made in the text and arrangement. In some points the author differs somewhat from the generally accepted views, but these perhaps have not been definitely settled. The work still remains in all respects a complete handbook of physiology.

